# Respect in the Eyes of Non-Urban Elders: Using Qualitative Interviews to Distinguish Community Elders’ Perspective of Respect in General and Healthcare Services

**DOI:** 10.3390/ijerph19042171

**Published:** 2022-02-15

**Authors:** Yu-Hsien Tseng, Yu-Ling Li, Shyuemeng Luu, Dih-Ling Luh

**Affiliations:** 1 Department of Public Health, Chung Shang Medical University, No.110, Sec.1, Jianguo N.Rd., Taichung 40201, Taiwan; piano51232@gamil.com (Y.-H.T.); cr24980013@gmail.com (Y.-L.L.); 2 School of Health Administration, Dalhousie University, Sir Charles Tupper Medical Building, 2nd Floor, 5850 College Street, P.O. Box 15000, Halifax, NS B3H 4R2, Canada; S.Luu@Dal.Ca; 3 Department of Family and Community Medicine, Chung Shan Medical University Hospital, No.110, Sec.1, Jianguo N.Rd., Taichung 40201, Taiwan

**Keywords:** community, the elderly, respect, healthcare services

## Abstract

This study aimed to describe the connotation of respect for community elders in daily situations, and discuss the elderly’s views on respect for healthcare services. A qualitative research design was conducted to interview elders from a non-urban area in Changhua, Taiwan. Study sites were Lukang and Ershui. A total of 52 people were interviewed, with an average age of 75 years old. Based on Grounded theory, the thematic analysis method was used to analyze data. This study found that respect from the perspective of the elderly can be divided into three categories: (1) verbal expression, (2) non-verbal behavior, and (3) behavior combined with appropriate language. We found that elders use the performance of healthcare service providers to discuss respect in the field of healthcare services. Respect can also be shown in the physical environment in healthcare settings. This study found that, for the community elders, respect is an individual’s subjective feelings regarding the process of interpersonal interaction. Compared to daily life, the respect of the elderly for the healthcare setting has increased the element of the environment. In addition, it was found that elderly people have lower expectations and requirements for respect in healthcare settings.

## 1. Introduction

As the proportion of older people increases globally, the development of an environment that meets the aspirations and needs of older people has become a major concern for society and public policy. Therefore, the World Health Organization has proposed age-friendly cities as a policy guideline for countries and pointed out that “respect” is the key to planning age-friendly cities. For policymakers, the essence of respect should be based on the viewpoint of the elderly, not just the professional perspective.

“Respect” is the foundation of human interaction [1] and one of the very important moralities in Chinese society. It was noted 1200 years ago in the Book of Han Shu, a Chinese history book of the Han Dynasty [2]. For elders, respect is also an important cultural expectation in different contexts. Therefore, the perceptions and the requirements of respect differ from one situation to another. Stephen L. Darwall (1977) divided respect into two: recognition respect and appraisal respect [3]. Recognition of respect means that as long as a person is part of our moral consideration, his or her existence has value and should be respected. This respect is equal for everyone. However, an individual is not only a body but also has multiple roles that interact with the environment and others, thus creating a variety of values. On the other hand, the second type of respect (Appraisal respect) is given based on a person’s abilities and values [3]. Very few studies have focused on the topic of respect for seniors. Ingersoll-Dayton and Saengtienchai conducted 79 focus groups in four Asian countries (Singapore, Taiwan, Philippines, Thailand) on elderly people aged 60 years or older and adults aged 30 to 55 years. The study concluded that both adults and seniors agree that “respect to the elderly” includes specific gestures and politeness, satisfying daily life needs of seniors, tradition, and etiquette, listening to advice, and obedience [4]. However, Ingersoll-Dayton failed to compare the difference of views on respect between adults and elders. Ronzi et al. used the Photovoice Methods to examine the perceptions of respect among elderly people living in Liverpool, UK. In Ronzi’s study, the participants were asked to take photographs of respectful people and things in their city of residence and then to explain the meaning of respect in the focus groups. It is important for elders to know who is respected and socially engaged in the city they live in. However, the perception of respect was not defined and did not conclude the contents of respect from seniors [5]. They failed to define what respect is and did not conclude the contents of respect from seniors. Later, Ronzi applied the same method to explore the feelings of the elderly toward an age-friendly city process. The authors found that seniors believe that respect and social tolerance are determined by both nature and social environment [6]. Although respect is an old issue, it is still worth further study.

The number of elders over 65 in Taiwan is increasing steadily and reached 16.2% of the population by the end of January 2021 [7]. The National Development Council estimates that Taiwan would be a super-aged society by 2025 [8] and seniors would account for one-fifth of Taiwan’s population. Although aspirations may be an overreach, the development of an environment that meets the needs of older people remains a major concern of society and public policy as the older population grows [9]. The most important aspect is “Active Ageing”. The World Health Organization (WHO) defined Active Ageing as the process of optimizing opportunities for health, participation, and security to enhance the quality of life as people age in 2002 [10]. WHO also proposed the concept of an Age-friendly City in 2007 to cope with policies, services, settings, and structures support needed for Active Ageing [11]. “Respect and Social Inclusion”, one of the eight dimensions, not only appears in infrastructure but human interaction as well. Previous studies verified that “Respect and Social Inclusion” correlates with other dimensions such as “Communication and Information” [12] and “Social Participation” [6]. Respect, therefore, is a core concept of an age-friendly city [11]. It is vitally important to plan an age-friendly city by apprehending the view toward respect and experiences.

Respect is not only a topic of daily life but a subject that medical and health service providers should pay attention to. As indicated by Beach: Respect is fundamental to all human interactions and especially important in healthcare, where its presence allows for some levels of patient dependency without fear of mistreatment or abuse [1]. Previous qualitative studies explored seniors’ responses toward healthcare services and found that the experiences of healthcare utilization play an important role, including health service utilization access, health service utilization, and service quality [13,14,15,16]. For instance, the following make the elderly sense disrespect including “waiting for services” and “passive attitude of healthcare service providers” [13], “careless healthcare service providers ” [14], and “healthcare providers do not listen to patients’ complaints” [15]. Koskenniemi’s study is one of the few qualitative research studies focused on respect [16] that tends to understand the view of hospitalized seniors regarding respect. The author found that respect includes, in admitted seniors’ viewpoint, “action of nurses”, “social support from relatives and friends”, and” information exchange in the ward” (including patients and caregivers) [16]. Although all of the above studies mentioned the respect of the elderly for the healthcare setting, respect was only one of their findings and not the purpose of the study. Moreover, these studies were all conducted in clinical settings. Therefore, understanding the respect of the elderly in the community (non-clinical) setting for healthcare services can help to provide more contextual and objective information.

Understanding the elderly’s views, experiences, and feelings of respect is an important subject but missing evidence-based support. Respect is a noun frequently used and heard nowadays, however, it is an abstract concept due to its vague definition. It would be appropriate to adopt a qualitative study design to examine an abstract concept because it can directly and deeply reveal the opinion and feelings of study participants. This study, therefore, adopts qualitative interviews to verify and compare the perspective of respect of the elderly from both daily life and healthcare settings.

## 2. Materials and Methods

Based on Grounded Theory, we conducted a face-to-face, in-depth qualitative interview to depict the elders’ perceptions of respect.

### 2.1. Background and Study Participants

Two townships of Lukang and Ershui in Changhua County were chosen in consideration of convenience. Changhua locates in central Taiwan with agriculture as its main industry [17]. There was a population of 1.26 million in Changhua in 2020; 16.6% were seniors over 65 years old, which was comparable to the overall senior distribution in Taiwan of 16.1% [8]. As for industry distribution in Changhua County, Lukang’s major industry includes commerce, tourism, and export processing [18]. Lukang is one of the earliest developed townships [19] with multiple cultural and religious historic sites [20] and a higher population density of 3104/km^2^. On the other hand, agriculture is the major industry in Ershui since it was developed late and with a lower population density of 1174/km^2^ [8]. According to the demographic statistics of Changhua County Government, the socio-demographic distribution of 65-year-olds and above in Lukang and Ershui is: more females than males (54.11% and 52.82%, respectively), and nearly 70% of the elderly have education level of elementary school and below (76.16% and 69.36%, respectively).

Study participants were recruited from local Community Care Stations (LCCS) that were established by local community offices and civil groups. The station invited residents to volunteer their effort to visit, phone-interview, and offer referral services to the elderly. Based on the needs of local seniors, the station also provided meal services and/or health promotion activities to elaborate community self-aid function through localized community care and offers a familiar environment for the elderly [21]. In Lukang, four community care stations participated: Yang Tze, Hsin Tze, Wan Hsin, and Nan Shi (Level-C station). There was only one in Ershui: the gym of Ershui Health Station (Level-C Medical station) [22]. Recruitment of participants for this study was not through advertising but the assistance of LCCS. The assistance provided by the LCCS leader included: (1) Asking for the willingness of the member to be interviewed. (2) Arranging a date and time for the interview with the senior who was willing to be interviewed. (3) Provide a space for the interview (usually at the LCCS’s office). (4) Introduce the researcher to the interviewee on the day of the interview.

This study estimated sample size through the Theoretical Sampling method including area (Lukang and Ershui), sex (male and female), education (elementary, junior high school, and senior high school). There were twelve categories and each category contained at least 3 participants. The cut-off point was set to duplicating interview information obtained and/or information saturation. The subject inclusion was the subject’s agreement of participation and the elderly over sixty-five years old.

Data collection was carried out from November 2020 to January 2021. Fifty-four elders agreed to be interviewed. A total of fifty-two people were interviewed after excluding two who were younger than sixty-five years old at the time of the interview, twenty-eight in Lukang and twenty-four in Ershui. The average age was seventy-five years old, and females were the majority (thirty-six).

### 2.2. Data Collection

Data were collected by four interviewers by using a semi-structured questionnaire. The study questionnaire was divided into two parts, starting with structured questions asking the elderly about basic demographic variables (sex, age, and education level) followed by a qualitative interview outline about respect (see Box 1 for details). For the qualitative interview, we asked only two questions: (1) could you please tell us what do you think respect means? As study participants were unable to answer the above questions or did not know how to describe them, interviewers would ask the elders to give examples. (2) After the study participants described their general perceptions of respect, they were then asked “What do you think is respectful in a healthcare setting?”. Similarly, the study participants were asked to give examples and explain the reasons. A complete outline of the qualitative interview is shown in Box 1.

Box 1 **Research Questionnaire: including structure questionnaire and qualitative interview outline.**

**Structured Questions**

Socio-demographic characteristics

1. Gender:☐ (1) Male ☐ (0) Female
2. Birth: year
3. Education Level:
    ☐(01) Elementary School ☐(02) Junior HighSchool
    ☐(03) Senior High School

**Qualitative Interview Outline**

[In general]

Main Question:

We all know that it is very important for people to respect each other, but we don’t know what our elders think about respect. So could you please tell us what do you think respect means?

(If the elderly person has difficulty answering the above questions, continued to ask)

would like to ask you or anyone else you know if they have ever experienced not being respected. Please describe the time and place of this experience.

 

[Healthcare Settings]

Main Question

What do you think is respectful in a healthcare setting (i.e., when using health services, when visiting a doctor)?

Continued

Is there a specific behavior that shows respect?

Continued

Have you ever experienced being disrespected in a medical setting? Why?

The interviewers received training one month before the interview launched. The training not only emphasized the accordance of interview technique but detailed every possible scenario and their solutions during the interview. Before the formal interview, the four interviewers conducted a pilot interview at the research site after the interview training and research ethics course to practice their interviewing skills and to test the applicability of the interview outline. A total of four elderly people who met the inclusion criteria were interviewed for this pilot study and were not included in the formal study sample.

The interview time varied due to the differences in response and answers of the elderly; it ranged from fifteen to sixty-five minutes with an average of thirty-three minutes.

This study passed the requirement set by the Institutional Review Board (IRB) of Chung Shan Medical University Hospital (CS2-20135, 2020). Before the interview, the purpose of study, study contents and all related right issues, and the reason for tape-recording of the interview were fully explained to all interviewees. Interviewees also signed two copies of the letter of agreement, participants and researchers each retaining one.

### 2.3. Data Analysis

This study was based on Grounded Theory [23] combined with thematic analysis [24] to conduct qualitative data analysis. It included the following steps: 1. Establishing verbatim manuscripts from interview contents; 2. Study verbatim manuscripts: the author examining verbatim manuscripts repeatedly along with another researcher; 3. Open coding: determining coding table that contains initial data after reading verbatim manuscripts; 4. Axial coding: theme finding (looking for a topic that represents verbatim manuscripts) and confirming the theme (both researchers confirm that there is enough data to support each theme); 5. Definition and naming the theme: defining and naming the theme that is supported by sufficient data; 6. Outcome searching: concluding the outcomes that explain the initial data.

## 3. Results

All of the following descriptions of respect are based on what the elders described. The inquiry did not specifically refer to elders, but rather to the broader concept of respect.

### 3.1. Respect in General

When asking the elderly “What do you think respect means?”. When analyzing the general view of respect of the elderly, we found that the majority of seniors do not convey subjectively how respect should be expressed during the interaction, and there was no “I” appearing in their responses. Less often taking himself/herself or a specific person as an example. The responses could be categorized in three dimensions after thematic analysis:

1. Respect is a way of verbal expression, for example, “gentle tone”, and “smile when speaking”.
  ***Participant:…soft voice and lower volume when speaking (L07M71)***
2. Respect is a non-verbal behavior including “listening”, “fairness”, “comprehending other people”, “do not cross the line”, “good rapport”, support”, “politeness”, and “filial piety”.
  ***Participant: Respect is…understanding each other, each other, right? Right, understand each other, it would be fine as long as we perceive what he thinks (U01F72).***
  ***Participant: Respect! It means do not interfere with other people. Other people do not interfere with us either (U27F70).***
  ***Participant: Respect for the view is mutual, you care about me and I will care about you so, should be mutual (U15M77).***
  ***Participant: Respect is…nothing particular, just be polite, with courtesy …(U24M71).***
  ***Participant:…they do respect me; two kids, one works at a railway station and the other takes care the store, (Interviewer: So, your kids all take care of you is a way of showing respect to you?) Participant: Yes, yes! (U06F80).***
3. Respect is a behavior combined with appropriate language including “responsiveness”, “conflict avoiding”, “praise”, and “greeting according to seniority in the family”.
  ***Participant: Respect is…(a short pause)…There were responses after you spoke (L12F75).***
  ***Participant: Respect means do not argue, do not quarrel (U08M75), do not offend other people, everyone should have mutual affection (L19F67).***
  ***Participant: Everyone respects me. …(Interviewer: So, how does everyone respect you?)…I told them that you are much older than I am, you cannot call me as “elder sister”…(L26F750).***

### 3.2. Respect in Healthcare Settings

The results of this section are based on the elders’responses to the second question of the Qualitative Interview Outline, i.e., “What do you think is respectful in a healthcare setting?”(see Box 1 for details). Through thematic analysis, four dimensions have emerged: (1) a way of verbal expression, (2) non-verbal behavior, (3) behavior combining with appropriate language, and (4) environment.

Overall, this study found that the elderly only mentioned the respect shown by healthcare providers when expressing their view of respect in healthcare settings. Without any other hints or follow-up questions, none of them mentioned their respect to either healthcare settings or healthcare providers.

#### 3.2.1. Respect Is a Way of Verbal Expression

According to seniors’ perceptions of respect in healthcare settings, seniors seem to have lower expectations or standards of respect in healthcare settings compared to respect in general situations. More than one senior has pointed out that as long as healthcare professionals speak in a kind, acceptable, or even “not-mean” manner, it is considered to be respectful.

***Participant: people will respect us more, the practitioners are all very courteous,…he treated us not in a mean way…(L01F70).***

***Participant: …It was OK! (Interviewer: Can you tell me why it was OK?) Participant: The tone of healthcare providers was acceptable! (L07M71).***

***Participant: respect me?…I felt he was kind when he spoke (L07M71),…Ya, both doctors and nurses spoke in a very nice way (L11F75).***

#### 3.2.2. Respect Is a Non-Verbal Behavior 

In terms of respect in healthcare settings, the statement of the elderly mainly involves the professional services of medical care providers. It indistinctly suggests that most elderly think healthcare services are medical services. As for respect in such a setting, it ranged from a broader and abstract concept of “overall service attitude” and “listening to the elderly” to “care services other than healthcare services”, and “attitude when offering healthcare services”. Furthermore, elders more directly use medical care outcomes as an indicator of respect, such as “providing expected medical care services” and “obtaining final health results”. In terms of non-verbal behaviors of respect, it is clear that the elderly have specific and diverse expectations of respect in healthcare settings, as opposed to verbal expressions of respect.

***Participant: I felt that everybody is very kind, with very good service, that’s it, this is the way to show respect to me (L25F68).***

***Participant: The doctor is more polite, more respectful of what you say, will listen to what you say, and will listen to whatever you want to say (U14M84).***

***Participant: They are very good, so they are very respectful. I had my tooth extracted, they were afraid that I would fall and would like to escort me to the station …the doctor asked a nurse to take me to a bus station (U22F73).***

***Participant: There is respect… that was a nurse, he was doing an ultrasound, when there was an issue, he said “let’s do it again, let’s do it again”, he would re-scan the spot thoroughly or ask you to come back another time (U24M71).***

***Participant: being respected… he would check what discomfort I described, prescribing medication for me (L17M84).***

***Participant: …cure me, that’s it…that’s respect…we wouldn’t visit again if he didn’t cure us, that’s impossible (U19M82)?***

#### 3.2.3. Respect Is a Behavior Combined with Appropriate Language

The study found the following in the current dimension: the elderly felt being treated with respect when healthcare providers offered services combined with appropriate language including “looking after”, “caring and greeting the elderly himself/herself”, “reminding and explaining during the process of healthcare services”, and “describing and explaining healthcare services”. We found that language was an important medium to convey respect to the elderly. Even for behavior, it needs to go along with language to show respect. As for the contents of language, the elderly stressed more of the personal caring aspect rather than an explanation of disease and services.

***Participant: respect…they would say ”sir, sit here and wait for a while”, that’s it, this is what I need. This is respect, what would it be otherwise? (L01F70). Participant: respect me…doctors are very nice nowadays when I entered, he invited me to sit near him, they all very kind (L03F74).***

***Participant: respect in this environment, they were nice to us, asked you “Did you eat This morning?...You need to pay attention when going out.” It made me feel this doctor is very nice (L15F73).***

***Participant: respect…doctors were very nice, currently, he knew how to treat me, “Hey, don’t eat sweet stuff!” He made fun of me, “You will get a needle if you eat sweet stuff again!” (Smiling), harassing me (L20F81).***

***Participant: Respect…what to say…the doctor pointed out the issue and how to treat it (U23M65).***

#### 3.2.4. Respect Is a Perception of Physical Environment Arrangement in Healthcare Settings

The study found that respect in healthcare settings is not only conveyed by healthcare providers, humans, it can also be delivered through the environment, such as “the distance between the elderly and healthcare providers” and “the privacy concern when receiving services”.

***Participant: Being respected… doctor sat closer to you, discussed the inner issue with you, a doctor sat across the table and the nurse was beside him/her, it felt like being examined (L04M76).***

***Participant: Respect for patients who come to see the doctor….you should offer privacy when they need it, not just open to everybody (U13F71).***

#### 3.2.5. The Elderly’s Perceptions of Respect in the Healthcare Setting Will Vary According to Sociodemographic Characteristics

After stratifying by sociodemographics, it is found the majority view of the elderly toward “respect in healthcare settings” was coherent in dimensions, but some examples in the dimension varied as follows:

1. Area difference: The elderly in Lukang care more about the verbal expression of healthcare providers. For example, ***Participant: respect me?…I felt he was kind when he spoke (L07M71),…Ya, both doctors and nurses spoke in a very nice way (L11F75).*** On the contrary, the elderly in Ershui are more concerned with “listening to the elderly”. For example, ***The doctor is more polite, more respectful of what you say, and will listen to what you say, and will listen to whatever you want to say (U14M84).***
2. Gender difference: Females stressed “offering care services other than healthcare services” and “being looked after”. For example, ***Participant: there is respect… that was a nurse, he was doing an ultrasound, when there was an issue, he said “let’s do it again, let’s do it again”, he would re-scan the spot thoroughly or ask you to come back another time (U24M71). Respect…they would say ”sir, sit here and wait for a while”, that’s it, this is what I need. This is respect, what would it be otherwise? (L01F70).*** Males, however, payed more attention to “obtaining final health results” such as ***“Participant: …cure me, that’s it…that’s respect…we wouldn’t visit again if he didn’t cure us, that’s impossible (U19M82)?”***.

### 3.3. The Comparison between “Respect in Daily Life” and “Respect in Healthcare Settings”

**Figure 1:**
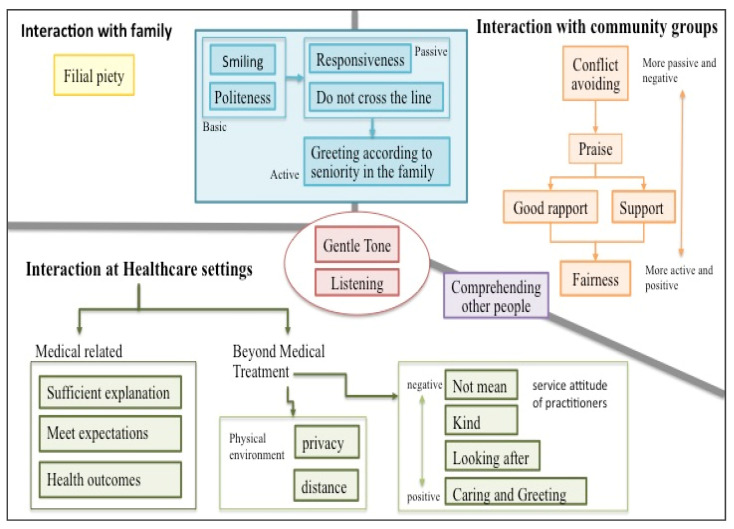
A diagram of respect in the eyes of the elderly depending on three situations.

Further analysis revealed that general daily life situations can be subdivided into family and community. Therefore, the following comparison of respect in different contexts is presented as (1) interactions with family members, (2) interactions with community groups, and (3) interactions in healthcare settings (depicted in Figure 1). he elderly’s comparison between “respect in daily life” and “respect in healthcare settings” is concluded as follows:

First, we found that two elements of respect occur in all contexts: “gentle tone” and “istening” The former is verbal and the latter is behavioral, both of which can be regarded as the most basic expressions of respect.

Secondly, in daily life situations, whether it is interactions with family members or community groups, there is a group of elements of respect as shown in the blue block in Figure 1. The elements of respect in the blue block include five items: (1) “miling”and “being polite” are basic behavioral respect; (2) “esponding”and “ot crossing the line”is more passive behavioral respect; and (3) “ddressing one’ peers” is a more active behavioral expression.

Third, the respect for interaction with community groups in daily life also appears in five elements. These elements of respect, in order of their proactive and positivity, are “conflict avoiding”, “praise”, “good rapport”, “support”, and “fairness”.

Fourth, there is a unique element of respect that exists only in the context of interaction with family members, and that is “filial piety”.

Fifth, there is a common element of respect in community groups and healthcare settings: comprehending other people.

Finally, there are nine elements of respect that appear only in healthcare settings, which can be divided into two categories: medical-related and beyond medical treatment. The former includes (1) sufficient explanation, such as reminding and explaining during the process of healthcare services, or describing and explaining the contents of healthcare services, (2) meeting expectations, such as providing expected medical care services; and (3) health outcomes, that is a cure or control for the disease. The respect for “beyond medical treatment” in healthcare settings can be further divided into two subcategories: the physical environment and the attitude of the practitioner. The respect related to the physical environment includes “privacy” (the privacy concern when receiving services) and “distance” (the distance between the elderly and healthcare providers). The respect element related to the attitude of the practitioner, which reflects seniors’ requests and expectations of the practitioner’s service attitude, was ranked from low to high: (1) not mean and not harsh; (2) kind or friendly; (3) looking after, such as arranging and greeting the elderly to be seated actively; and (4) caring for and greeting the elderly, such as reminding the elderly of traffic safety when leaving.

## 4. Discussion

This paper used the question “What do you think respect means?” to interview elders to understand their perspectives on respect. The qualitative analysis was used to construct the connotation of respect from the elder’s perspective. Some elders gave examples to illustrate their views on respect, and these examples included their own experiences and their observations. Therefore, respect in the eyes of the elders is a feeling of interpersonal interaction, which implies how they should behave when interacting with each other, and also includes self-requirement and expectation of the behavior of others. Furthermore, elders’ subjective perceptions of respect are influenced by the culture and traditions of the region in which they live. Therefore, the results of this study are not only the elders’ experiences of respect but also the elders’ overall perceptions of the concept of respect.

### 4.1. Respect in the Eyes of the Elderly

In terms of respect in daily life, this study found that the elderly are familiar with it and they do possess their way of thinking, which is congruent with previous studies. In terms of respect in daily life, this study found that the elders were familiar with the term respect and had their ideas about it, which is consistent with Ronzi’s studies [5]. Overall, it can be summarized as “For the elderly, respect is a subjective feeling that individuals have about the process of interpersonal interaction, which is shaped through mutual verbal expressions, non-verbal behavior, and behavior combined with appropriate language.” Overall, it can be concluded that respect is a subjective perception regarding human interaction and is shaped by verbal expression, non-verbal behavior, and behavior combined with appropriate language. This study found that previous studies rarely mentioned that the elderly consider that verbal expression is a way of respect. The dimension of non-verbal behavior in respect, however, is similar to what Ingersoll-Dayton and Saengtienchai attained [4].

In terms of respect in health services, it can be summarized as “For the elderly, respect in healthcare settings is a subjective evaluation of the process, the provider, and the environment in which the service is provided, and is composed of verbal expressions, nonverbal behaviors, behaviors combined with appropriate language, and the physical environment of the healthcare setting.” The attitudes and behaviors of health service providers mentioned by the elderly in various aspects were similar to previous studies. Take the study in Finland as an example, admitted elders believe the following are ways to show respect: nurses should respond to patients’ questions/requests, listen to the elderly, and offer healthcare information [16]. Beach’s study, conducted in the US, indicated that African Americans consider the following are also ways of respect: doctors listen to patients’ complaints and do not deny them, show care to patients, and explain medical-related questions honestly [1]. A study conducted in Ghana indicated that low-income seniors think it is disrespectful for healthcare providers not to listen to patients [15]. The elderly value the attitude of healthcare providers when providing healthcare services, and even make a judgment based on healthcare providers’ attitude whether they have received the respect or not. A possible explanation is that elderly link the passive attitude of healthcare providers to what they believe is “Ageism” [13].

The issue of respect in the environment of healthcare settings has not been found in daily life. The elderly think “ensuring privacy” in healthcare settings is a way of respect, which corresponds to an England study: elders believe “maintaining privacy” is the main factor of care with dignity [25]. This study, however, found that the elderly think it is a way to show respect through the distance between healthcare providers and the elderly. There is no similar result that other studies found though; the authors think a possible explanation could be:

3. Compared to general situations, it is easier to present a clearer and different perspective on a specific situation. A healthcare setting is not only a more specific context for the seniors but also a place to treat their health problems. Interpersonal interactions in this setting include both general and therapeutic levels. In addition to interpersonal interaction, it also includes the perception of environmental arrangements. From the elderly’s statement, the expectation of respect for the healthcare setting is implied: I am not just a health problem to be treated, but a person.
4. The elderly believe subconsciously that respect in healthcare settings contain his/her expectation in utilizing healthcare services including space planning. The closer spatial distance seems to make the elderly feel more respected. This result is consistent with the psychological view that spatial distance reflects psychological distance, and that psychological distance can be reduced by shortening spatial distance [26].

### 4.2. The Subjectivity of Respect: Respecting Others or Being Respected

Is respect in the eyes of the elders respect for others or being respected? We found that: (1) In daily life, respecting others and being respected are two sides of the same coin, and (2) seniors are more concerned about “being respected” in the healthcare setting. The discussion is as follows.

#### 4.2.1. In Daily Life, Respecting Others and Being Respected Are Two Sides of the Same Coin

We found that respect in the eyes of the elders manifested itself in the interactions of daily life. Although respect can theoretically be divided into active and passive aspects, the elders seemed to view respect for others and being respected as two sides of the same coin. The elders usually discussed it without a subject word or outlined it as “mutual”. For example, responding to each other in conversation. Or a polite interaction in which “everyone is polite”. Or “don’t cross the line” to describe respect for each other.

Ingersoll-Dayton [4] used adults and elders as study participants in four Asian countries and conducted many focus groups to ask “How do people show respect for the elderly?”. They summarized the following characteristics regarding respect for the elderly: gestures and manners, tokens, customs and rituals, asking for advice, and obedience [4]. We can find that the results of Ingersoll-Dayton’s study also contain active and passive perspectives, for instance, “accepting recommendations and obedience” is a passive way of respecting elders. They found that respect in daily life is mutual and not just offering or receiving.

#### 4.2.2. Seniors Are More Concerned about “Being Respected” in the Healthcare Setting

Unlike respect in everyday life, we found that seniors’ perception of respect in healthcare settings is unidirectional. All of the narratives in our findings were about respect of the delivery of healthcare services, and none of them mentioned the respect that patients have for the healthcare setting or the provider. In terms of respect, healthcare providers are contributors and the elderly are receivers. This result is consistent with previous studies in other countries. Past research on respect in healthcare settings has focused on the respect of healthcare providers for their patients. For example, in a study in South Africa, it was found that the elders perceived the negative attitude of the medical staff [13] or lack of concern for patients as disrespectful [14]. Ghana’s study found that seniors considered it disrespectful for healthcare providers to fail to listen to their complaints about health problems or to ignore their expectations about treatment options [15]. A Finnish study of hospitalized seniors found that seniors perceive nurses to be respectful by being courteous, listening patiently, providing medically relevant information, being responsive, and providing basic care [16]. To date, no studies have been found that focus on patient respect for the healthcare system. The unidirectionality that we propose in this paragraph means that respect for the healthcare setting seems to be focused only on one direction of the provider to the patient. This may be due to a long history of inequality in the knowledge and power structure of the physician–patient relationship. Most research on the physician–patient relationship has focused on how physicians should respect their patients, while patient respect for physicians seems to be underemphasized because it is a universal fact.

### 4.3. The Discussion of the View for the Elderly toward Respect in Healthcare Settings

#### 4.3.1. The Area Differences in the View toward Respect in the Healthcare Setting

This study found some slight area differences in the perceptions of respect in health service settings among the elderly. (1) Those who perceived respect as a verbal expression were all from the respondents in Lugang. These seniors seemed to have lower expectations or standards of respect in healthcare settings compared to respect in general situations. (2) Those who thought that listening to patients was a form of respect were the respondents from Ershui. Ershui’s seniors could provide real examples to express what they consider to be respectful of healthcare. For example, the kind greetings from doctors, the care for the elderly in their daily lives outside of medical treatment, etc.

This could be attributed to different interviewing locations: a community care station in Lukang and the gym in a health station in Ershui. When being asked about “respect in healthcare settings”, the majority of the elderly in Ershui replied “The director in the health station is very nice” in the beginning. They replied more once the interviewer asked deeper. The elderly in Lukang shared their previous experiences instead of their impression of specific healthcare provided. The elders mentioned that the respect of healthcare providers is shown through careful examination during treatment. It could be due to the variation of care-seeking location, health station for Ershui’s elders, and hospitals or clinics for the elderly in Lukang.

#### 4.3.2. The Gender Differences in the View toward Respect in the Healthcare Setting

There are differences between male and female seniors toward the view of respect in the healthcare setting. Female elders felt being respected through “offering care services other than healthcare services” (non-verbal behavior) and “looking after” by healthcare providers (behavior combining with appropriate language). For example, while waiting for a consultation, the nurse asks if the patient needs a drink of water or to sit down for a break. During the consultation, the doctor will take care of the elderly’s daily life, such as whether they have eaten breakfast or not. After the visit, the doctor will ask a nurse or volunteer to accompany the elder to the station. Male elders, however, believed that respect is “obtaining final health results” (non-verbal behavior). For example, curing a disease is respect.

From the above interpretation, it seems that female elders are more process-oriented, while male elders are more outcome-oriented. Compared to males, females’ perception of respect originates through multiple sources including language and behavior. Previous studies of respect concentrated on females and very few of them focused on males. In Janevic’s study, women hesitated to seek care because they did not feel they were being treated respectfully and thought they were discriminated against [27]. In Kujawski’s study, disrespectful incidents can affect a maternal’s satisfaction with her medical care [28]. In Tickle’s study, the higher the satisfaction of maternal women to midwife students, the higher the respect felt [29]. Combining results from both previous studies and the current research suggests that women are sensitive to respect and it further influences their health and medical service utilization.

#### 4.3.3. The Characteristics of Respect in Healthcare Settings

The elders think gentle tone, responding, and listening are ways of showing respect in both daily life and healthcare settings. The elders seem to have a lower requirement for respect in healthcare settings in that respect is expressed in a manner of acceptable, not mean, or not harsh tone. In healthcare services utilization, some elders raised the view that respect with no excess expression is an acceptable tone for providers.

It can be seen that the elders possess a similar view of respect in both daily life and healthcare settings. They play a passive receiver role in healthcare settings and pay less attention and care to respect. In the process of medical sociology development, the roles of medicine and society evolve constantly. Medicine is not merely a science, it can sculpt a society or vice versa [30]. This indicates that elders have fewer expectations of receiving respect in healthcare settings when compared to community settings, as the need to give respect to health professionals intersects with the need to give elders respect. Within healthcare settings, elders are also in a dependent relationship with their treating health professionals, and this may therefore demand that they give more respect than they expect to receive. In the development of medical sociology, the roles of medicine and society are constantly evolving, and medicine is not just a science but can shape society or be shaped by society.

The possible explanation could be due to the gap between patients and healthcare professionals and the latter plays an active role most of the time. The doctor–patient relationship produces an asymmetrical power structure due to the capability gap and makes users ask for less in healthcare services [31]. It could be also caused by “systematic conversation” and a “top to bottom doctor–patient relationship” that facilitates the passive role of care seekers while communicating with providers [32]. Another study revealed that patients’ voice is ignored and physicians are used to interacting with patients through medical professional framework while analyzing via “active-passive model” to investigate doctor–patient interactions [33]. Hence, asymmetry is created by an imbalance of knowledge and power that causes active and passive roles in doctor–patient interactions.

Based on the above discussion, healthcare practitioners and health policies have recently attempted to develop and promote measures to facilitate physician–patient communication, such as shared decision-making. The literature [34] suggests that shared decision-making can enhance patient engagement in the treatment process, which in turn improves outcomes and patient satisfaction. At the same time, healthcare professionals are required to respect the opinions and feelings of patients, and as a result, patients’ perceptions of respect in the healthcare setting change as well. Furthermore, our study found that in addition to condition-specific discussions, there are non-medical measures that can improve the feeling of respect in healthcare settings for the seniors, such as more personal greetings and care outside of treatment, or changes to the environment in which they are seen.

### 4.4. Study Limitation

#### 4.4.1. A General and a Short Outline of the Interview

Some of the respondents were brief in expressing abstract concepts (e.g., respect). Therefore, this study was conducted through constant encouragement and gentle probing by the interviewers to obtain richer information. However, to understand the perceptions of respect among the elderly without any specific orientation, the interview protocol was not pre-designed to include any specific orientation. For example, respect in everyday life was not specifically asked about, such as family or community groups. Similarly, when the elderly were asked about respect in health services, they were only asked about their perceptions of respect in a broad sense, without specifically including environmental factors and interpersonal interactions, which may have prevented them from giving focused responses. Therefore, the various aspects of respect and the conceptual framework found in the findings of this study all emerged from the information provided by the elderly interviewees. If future research would like to know the perceptions of elders about specific dimensions of respect, it is recommended to ask more specific questions, such as: “What are the things that the elderly feel respected for in the healthcare setting? Do they respect each other during interactions with healthcare providers?”

#### 4.4.2. Participants Were Limited by Senior Attendees in Community Care Stations

Firstly, because the elderly are more accustomed to community care stations and trust them more, the participants were introduced by community care stations. Therefore, elderly people who were not involved in community care stations were less likely to be participants in this study. Secondly, because qualitative interviews require informative participants and ethical considerations of informed consent, this study invited people to participate in the study through the assistance of a community care station. This recruitment channel may have biased the study results in terms of health effects: that is, seniors who participated in community activities are healthier than those who do not. The elderly who participate in the community care station may have different perceptions of respect. The generalizability of the results is limited by this group of community care center participants. Furthermore, since social and cultural backgrounds may have an impact on the elderly’s perceptions of respect, this study invited the elderly from the same county as the research subjects, whose living environment and cultural backgrounds may be similar, so the results of this study cannot be inference. Therefore, in the future, it is necessary to invite the elderly from different backgrounds as research subjects to explore the perceptions of respect among elders from different social and cultural backgrounds. In addition, there are local variations in healthcare resources and healthcare systems, which may affect the use of health services for the elderly, and these experiences of use may also affect their perceptions of respect. Therefore, it is suggested that health system factors could be taken into account in future studies.

### 4.5. Implications

#### 4.5.1. Understanding Seniors’ Perceptions of Respect Is Useful for Implementing Age-Friendly Cities

The World Health Organization (WHO) advocates for the promotion of age-friendly cities [11] around the world, where respect is not only one of the aspects, namely respect and inclusion, but also a key spirit in building the other aspects, especially for “social participation”, “civic participation and employment”, “communication and information”, and “community support and health”. Understanding local seniors’ perceptions of respect and providing a physical and social environment where seniors are respected will be the cornerstone of a successful age-friendly city. If respect as portrayed by elders represents their expectations, our study found that the respect that elders expect is very simple: it is expressed in what they say and do during interpersonal interactions, whether it is with their family, in the community, or a healthcare setting. Therefore, attention should be paid to interpersonal interactions at any opportunity when planning, designing, or providing age-friendly related services, as the language and expressions of service providers in their interactions with seniors may be a key factor for seniors to feel respected.

#### 4.5.2. It Is Recommended That Healthcare Providers should View the Patient as a Whole Person, Not as a Health Problem to Be Remediated

This study found two characteristics of elderly people’s perceptions of respect in healthcare settings that are worth highlighting: (1) Compared to respect in general life situations, respect in healthcare settings seems to be passively received by the elderly. (2) Respect in healthcare settings is perceived by the elderly to include many non-therapeutic components. It seems to be implied that (1) in their role as patients, the elderly seem to take their respect for healthcare providers for granted and do not need to say so specifically. (2) The elderly seem to want to be seen as a person and not just a disease to be dealt with. Therefore, based on the findings of this study, it is recommended that healthcare providers should view the patient as a whole person, not as a health problem to be remediated.

#### 4.5.3. Recommendations for Future Research

For this study, in-depth personal interviews were used to collect qualitative data to avoid interference from others and privacy concerns. It is suggested that future research could be conducted in focus groups, for example, because of the difference in responses between individual interviews and group interactions.

The elderly respondents were recruited through the assistance of the community care station, which may have resulted in a lack of elderly who did not participate in community activities. Therefore, it is suggested that future studies may target those who did not participate in any community activities to find out whether they have different views on respect.

This study attempted to understand the elderly’s perceptions of respect through broader, non-induced interviews. Not only was it found that the meaning of respect was illustrated in different contexts, but the perceptions of respect also varied from scenario to scenario. Therefore, future studies may design more follow-up questions to obtain more specific perceptions of respect according to the researcher’s concerns. For example, respect in a healthcare setting can be subdivided into questions about people (e.g., doctors, nurses, pharmacists), procedures (e.g., registering, waiting for a consultation, seeing a doctor, receiving medication, checking out), and the physical environment (e.g., waiting room, indicators, doctor’s consultation room, pharmacy).

Finally, to understand the distribution of elderly people’s perceptions of respect, we recommend designing a structured questionnaire based on the findings of this study and conducting a quantitative survey with a larger representative sample.

## 5. Conclusions

This study found that, through in-depth interviews, for the community elders, respect is the individual’s subjective perception regarding the process of interpersonal interaction. It is shaped by verbal expression, non-verbal behavior, and behavior combined with appropriate language. When inquiring more about respect in healthcare settings, the elderly believe respect in healthcare settings is a subjective assessment of healthcare providers while offering healthcare services. It is achieved through healthcare providers’ verbal expression, non-verbal behavior, behavior combined with appropriate language, and the physical environment of healthcare settings. Respect can be perceived differently depending on the person and situation with whom you are dealing. The ways of showing respect can range from active to passive. In this study, it was found that gentle tone and listening are the core concepts of respect in all three contexts (interaction with family, community groups, and healthcare settings). In conclusion, the perceptions of respect vary along with scenarios and controllability. At the same time, these results could offer healthcare practitioners a useful hint to provide more respectful services which fit the elders’ implicit expectations. In addition, the results of this study will help one to understand the elderly and promote intergenerational communication as well as a more harmonious doctor–patient relationship.

## Data Availability

All the data relevant to the study are included in this article. For further questions about the data, please contact the corresponding author (luh@csmu.edu.tw).

## References

[1] Beach M.C., Branyon E., Saha S. (2017). Diverse patient perspectives on respect in healthcare: A qualitative study. Patient Educ. Couns..

[2] (2021). Chinese Words, Respect. ChineseWords.org, R.O.C (Taiwan). https://www.chinesewords.org/dict/95538-35.html.

[3] Darwall S.L. (1977). Two Kinds of Respect. Ethics.

[4] Ingersoll-Dayton B., Saengtienchai C. (1999). Respect for the elderly in Asia: Stability and change. Int. J. Aging Hum. Dev..

[5] Ronzi S., Pope D., Orton L., Bruce N. (2016). Using photovoice methods to explore older people’s perceptions of respect and social inclusion in cities: Opportunities, challenges and solutions. SSM Popul. Health.

[6] Ronzi S., Orton L., Buckner S., Bruce N., Pope D. (2020). How is Respect and Social Inclusion Conceptualised by Older Adults in an Aspiring Age-Friendly City? A Photovoice Study in the North-West of England. Int. J. Environ. Res. Public Health.

[7] Directorate General of Budget, Accounting and Statistics (2021). Population and Housing Census.

[8] National Development Council (2020). Trend in the Percentage of Elderly Population (In Chinese).

[9] Buffel T., Phillipson C., Scharf T. (2012). Ageing in urban environments: Developing ‘age-friendly’cities. Crit. Soc. Policy.

[10] WHO (2002). Active Ageing: A Policy Framework.

[11] WHO (2007). Global Age-Friendly Cities: A Guide.

[12] Liu L.C., Kuo H.W., Lin C.C. (2018). Current Status and Policy Planning for Promoting Age-Friendly Cities in Taitung County: Dialogue Between Older Adults and Service Providers. Int. J. Environ. Res. Public Health.

[13] Kelly G., Mrengqwa L., Geffen L. (2019). They don’t care about us: Older people’s experiences of primary healthcare in Cape Town, South Africa. BMC Geriatr..

[14] Naidoo K., van Wyk J. (2019). What the elderly experience and expect from primary care services in KwaZulu-Natal, South Africa. Afr. J. Prim. Health Care Fam Med..

[15] Agyemang-Duah W., Arthur-Holmes F., Peprah C., Adei D., Peprah P. (2020). Dynamics of health information-seeking behaviour among older adults with very low incomes in Ghana: A qualitative study. BMC Public Health.

[16] Koskenniemi J., Leino-Kilpi H., Suhonen R. (2013). Respect in the care of older patients in acute hospitals. Nurs. Ethics.

[17] Changhua County Government Department of Agriculture (2015). Changhua Combines Industry, Government, and Academia for the First Time in Cross-Region Integration to Organize the Agricultural Innovation Institute Forum (In Chinese).

[18] (2018). Ministry of Labor Taichung-Chunghua-Nantou Regional Branch, Workforce Development Agency. R.O.C (Taiwan). https://www.wda.gov.tw/en/cp.aspx?n=D2DEEE3B0F86AA88&s=575F716EF3A537C0.

[19] (2021). Wikipedia, Changhua County. R.O.C (Taiwan). https://zh.wikipedia.org/zh-tw/%E5%BD%B0%E5%8C%96%E7%B8%A3.

[20] (2021). Wikipedia, Lukang Township County. R.O.C (Taiwan). https://zh.wikipedia.org/wiki/%E9%B9%BF%E6%B8%AF%E9%8E%AE.

[21] Social and Family Affairs Administration Ministry of Health and Welfare, Community Care Site (2021). Ministry of Health and Welfare, Taipei City, R.O.C (Taiwan). https://ccare.sfaa.gov.tw/home/other/about.

[22] (2021). Changhua County Government Department of Social Affairs, Senior Citizens Section Community Care Site. Changhua County Government, Changhua County, R.O.C (Taiwan). https://social.chcg.gov.tw/07other/other01_con.asp?topsn=711&data_id=11084.

[23] Birks M., Mills J. (2015). Grounded Theory: A practical Guide.

[24] Braun V., Clarke V. (2006). Using thematic analysis in psychology. Qual. Res. Psychol..

[25] Cairns D., Williams V., Victor C., Richards S., Le May A., Martin W., Oliver D. (2013). The meaning and importance of dignified care-findings from a survey of health and social care professionals. BMC Geriatr..

[26] Williams L.E., Bargh J.A. (2008). Keeping one’s distance: The influence of spatial distance cues on affect and evaluation. Psychol. Sci..

[27] Janevic T., Sripad P., Bradley E., Dimitrievska V. (2011). There’s no kind of respect here A qualitative study of racism and access to maternal health care among Romani women in the Balkans. Int. J. Equity Health.

[28] Kujawski S., Mbaruku G., Freedman L.P., Ramsey K., Moyo W., Kruk M.E. (2015). Association between disrespect and abuse during childbirth and women’s confidence in health facilities in Tanzania. Matern. Child Health J..

[29] Tickle N., Gamble J., Creedy D.K. (2020). Women’s reports of satisfaction and respect with continuity of care experiences by students: Findings from a routine, online survey. Women Birth.

[30] White K. (2016). An Introduction to the Sociology of Health and Illness.

[31] Tripathi J., Rastogi S., Jadon A. (2019). Changing doctor patient relationship in India: A big concern. Int. J. Commun. Med. Public Health.

[32] Mei-Hui T., Feng-Hwa L. (2001). The Use of Appropriate Follow-up Responses to Patients as a Linguistic Way of Facilitating Doctor-patient Communication. J. Med.Educ..

[33] Shu-Chen K., Hsiao-Chu W. (2016). Effect of Doctor-Patient Interactions on Medication Use for Insomnia Patients. J. Humanit. Soc. Sci. Med..

[34] Eliacin J. (2015). Factors influencing patients’ preferences and perceived involvement in shared decision-making in mental health care. J. Ment. Health.

